# Recovery of platelet‐rich red blood cells and acquisition of convalescent plasma with a novel gravity‐driven blood separation device

**DOI:** 10.1111/tme.12830

**Published:** 2021-11-10

**Authors:** Dion Osemwengie, Johan W. Lagerberg, Richard Vlaar, Erik Gouwerok, Mya Go, Arno P. Nierich, Dirk de Korte

**Affiliations:** ^1^ Clinical Department, HemoClear BV Zwolle The Netherlands; ^2^ Department of Blood Cell Research, Sanquin Research Amsterdam The Netherlands; ^3^ Landsteiner Laboratory, Amsterdam UMC University of Amsterdam Amsterdam The Netherlands; ^4^ Department of Anesthesiology and Intensive Care, Isala Zwolle The Netherlands

**Keywords:** autologous blood, autologous blood technology, blood filter, blood separation, cell salvage, cell salvage technology, convalescent plasma, platelet‐rich RBC

## Abstract

**Objectives:**

Our objectives were to determine the separation characteristics and blood product quality of a gravity‐driven microfiltration blood separation system (HemoClear, The Netherlands).

**Background:**

A range of centrifugal blood separation devices, including intraoperative cell salvage devices (cell savers) and apheresis machines, are available to assist in preparing both allogenic and autologous blood products. These devices are expensive to operate and require extensive training.

**Methods and Materials:**

Nine whole blood units were collected under standard conditions and analysed for haematological parameters, thromboelastographic properties, platelet morphology and activation, and red blood cell (RBC) deformability and morphology. Three whole blood units were separated by means of the HemoClear device, into a liquid and cellular component. The cellular component was diluted with SAGM and cold stored for 14 days. To simulate cell salvage six whole blood units were diluted with isotonic saline, followed by multiple HemoClear separation rounds.

**Results:**

The recovery of both RBCs (100 ± 1.6%) and white blood cells (99 ± 4.5%) after undiluted filtration were very high, while platelet recovery was high (83 ± 3.0%). During the filtration, and cold storage after filtration storage both the non‐deformable RBC fraction and the RBC maximum elongation remained stable. Parameters of thromboelastography indicated that platelets remain functional after filtration and after 7 days of cold storage. In the cell salvage simulation the total protein load in the cellular fraction was reduced by 65 ± 4.1% after one washing round and 84 ± 1.9% after two consecutive washing rounds.

**Conclusion:**

The novel blood filter studied effectively separates whole blood into diluted plasma and platelet‐rich RBCs. Moreover, the device effectively washed diluted whole blood, driving over 80% of proteins to the liquid component.

## 1 INTRODUCTION

Patient blood management is a cornerstone of healthcare.^1^ Each year over 118 million blood donations are collected (WHO, Blood safety and availability factsheet^2^), but autologous blood transfusion, including cell salvage (i.e., re‐infusion of patients' own lost blood) is used increasingly.^3^ Both in processing of allogeneic and autologous blood, centrifugation‐based technology is the gold standard.

Based on differences in density and buoyancy, centrifugation‐based technologies effectively separate or wash blood components; whole blood (WB) donations are separated to facilitate optimal storage of, and clinical need for, the individual blood components.^4^ Moreover, use of apheresis blood component isolation is growing rapidly, and mostly relies on centrifugal technology as well.^5^, ^6^ Autologous shed blood is washed by so‐called ‘cell savers’ in order to reduce non‐cellular contaminants. Although robust and reliable, centrifugation‐based technology entails various disadvantages. As a result scientists have begun to explore centrifugation‐free washing methods.^7^

Various studies have indicated that washing of red blood cells by centrifugation‐based technology reduces the blood cell quality. Haemolysis and sublethal injury have been shown to occur immediately after washing and to continue during the afterward storage^8^, ^9^ indicating that the centrifugation induced shear stress increases RBCs fragility. In addition, the erythrocytes' ability to change shape, referred to as deformability, seems to be negatively affected by centrifugal washing.^10^ Apheresis was found to cause platelet activation, and erythrocyte complement disposition and antigen alterations.^11^, ^12^

Aside from blood quality limitations, blood processing centrifuges with the necessary disposables represent high capital expenditure and operational cost.^13^, ^14^, ^15^ Hence in the emerging world, where cost‐effectiveness is imperative, the centrifugal devices are largely inaccessible.^16^ Moreover, the ponderous design and need for a stable electricity source prevents use of centrifugal technology in resource‐poor, rural and military settings.^17^ In the settings where centrifugation‐based technologies are not available, not practical or not affordable, alternative non‐centrifugal blood separation technologies are invaluable. Here we describe an explorative evaluation of a novel blood microfilter (HemoClear BV, Zwolle, The Netherlands) for the use of WB separation and washing of shed blood.

**FIGURE 1:**
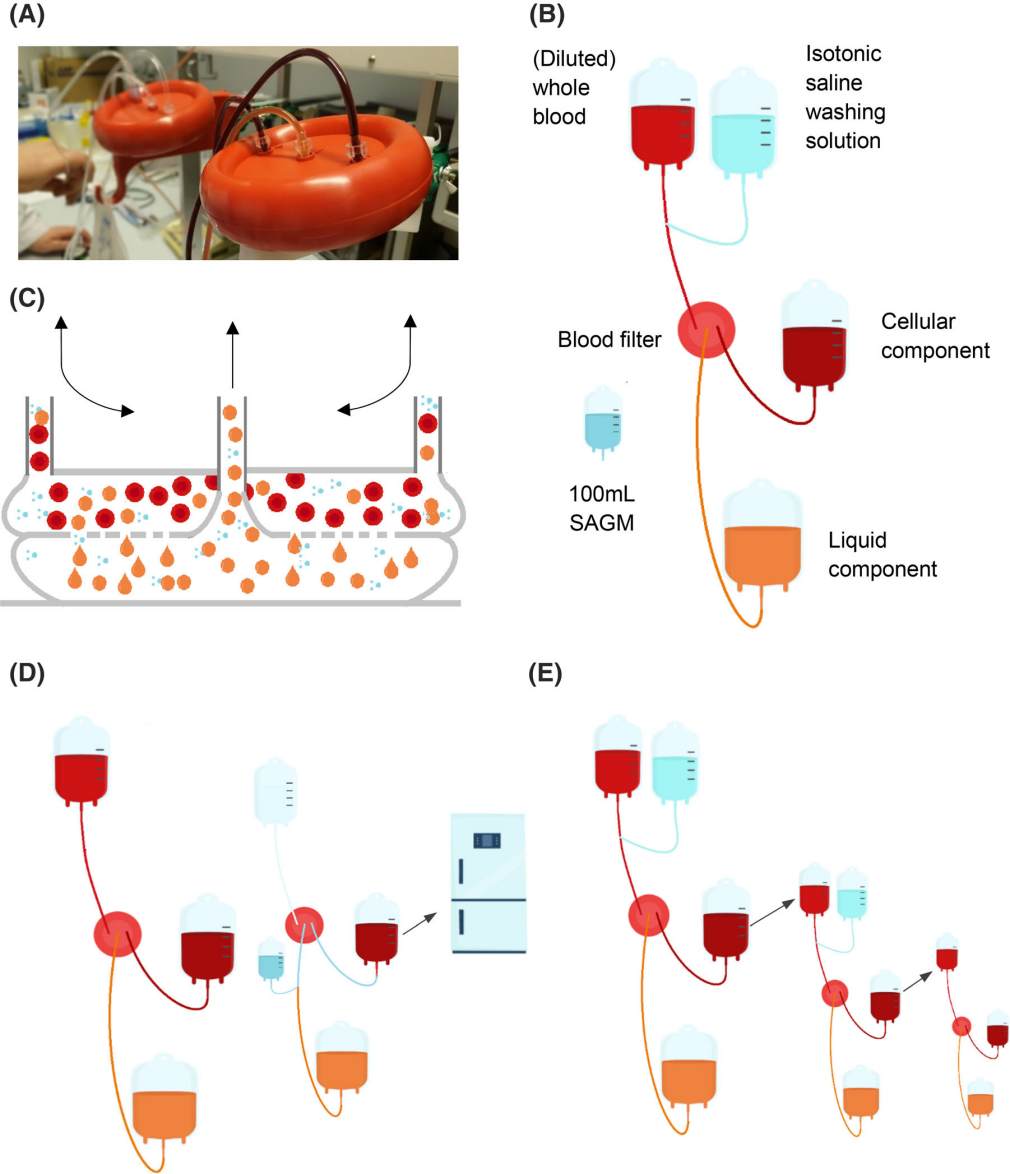
Blood separation system setup. (A) The HemoClear device while filtering. (B) Blood separation system in which the HemoClear filters is centralised between two blood bags and a filtrate bag that contains the liquid component. To the initial blood bag, washing solution can be added by means of three‐way tubing. (C) HemoClear cross‐flow microfiltration technology. (D) Cell salvage simulation protocol. (E) Blood separation protocol

Because this device does not comprise of any electrical components—flow is established merely by gravity and capillary action—mechanical and shear forces are anticipated to be lower compared to centrifugal machines. To explore its use in various settings, the filter was evaluated for both WB separation and for washing of diluted WB (simulated salvage of shed blood). Based on the patented cross‐flow micro‐filtration technology (Figure 1C), blood cells should be retained by the filter, while solutes and plasma are washed out. Due to the relatively low shear forces, we hypothesised cellular morphology, erythrocyte deformability and platelet function to be unaltered in the filtration processes.

## 2 METHODS

### 2.1 Blood collection

All (non‐remunerated) volunteer blood donors met standard donation criteria and gave their written, informed consent, in accordance with the institution's guidelines and practices. This study was approved by the institutional medical ethical committee, in accordance with the standards laid down in the 1964 Declaration of Helsinki. A total of nine WB units, 500 ml ± 2%, were collected in quadruple, bottom‐and‐top collection systems containing 70 ml of citrate–phosphate‐dextrose (CPD, Fresenius Kabi, Emmer Compascuum, the Netherlands) at the Sanquin Blood Center (Sanquin, Amsterdam, The Netherlands). The WB units were placed on butane‐1,4‐diol cooling plates (Compocool, Fresenius Kabi) to allow their temperatures to remain at 20–24°C until the start of the filtration study protocol.^18^ The day of blood collection was designated as day 0 of the study; the filtration study protocol was initiated at around 16 h after collection.

### 2.2 Separation of WB by means of a gravity‐driven microfilter

Separation of WB was performed with the HemoClear device (HemoClear BV) (Figure 1A). According to the device manufacturer, the device yields two filtration products; concentrated blood cells (referred to as cellular component) and the plasma (referred to as liquid component) (Figure 1B). The filter was used in a two‐blood bag system that was primed with isotonic saline prior to use. Due to cross‐flow technology the RBCs can enter the filtration device from either of the two inlet ports (Figure 1C). This feature allows for the filtration system to remain closed during consecutive filter rounds. Being disposable, a HemoClear system can be used to salvage multiple units of autologous blood provided that these were shed by the same patient. For the next patient a new HemoClear system should be used. Similarly, in this protocol a new HemoClear system was used for each new unit of (diluted) WB.

The device performance was evaluated in two protocols; a WB separation protocol and a washing protocol. The separation protocol (Figure 1D) was performed with undiluted WB and was intended to separate the cellular component from the liquid component. The washing procedure was performed with diluted WB to mimic shed blood (Figure 1E). This procedure was intended to remove unwanted solutes from the cellular component.

#### 2.2.1 Separation of WB

Three WB units were filtered through a HemoClear device by gravity (i.e., for each unit a new HemoClear device was used). Upon completion of the filter run, 100 ml of SAGM (Feresenius Kabi) was added to the cellular component by means of a backflush through the filter via an inlet in the liquid product line (Figure 1D). Both the liquid and cellular components were analysed for composition and quality immediately after the separation procedure. The cellular component was stored for 7 days at 2–6°C and analysed again.

#### 2.2.2 Washing of WB

Six half units of WB (around 300 ml) were diluted with a calculated volume of 0.9% NaCl (around 300 ml) to achieve 600 ml of diluted WB with an haematocrit (Ht) of 20%.

The six diluted WB units were subjected to separation by six HemoClear devices, driven by gravity. Upon completion, 300 ml of 0.9% NaCl was added to the cellular component and a second filtration round was performed. Consecutively, the second cellular component was subjected (i.e., without fluid addition) to a third filtration round to concentrate the cellular compound.

### 2.3 Measurements of blood component quality

#### 2.3.1 Volume

The volume of blood components was calculated from the net weight and the specific gravity: 1.026 g/ml for plasma, 1.100 for RBCs, and 1.006 for SAGM. Based on the haematocrit values, volumes for WB, diluted WB and RBCs in additive solution were determined.

#### 2.3.2 Haematological parameters

Haematological parameters (cell count, total haemoglobin concentration, haematocrit and mean corpuscle volume) were obtained using a haematology analyser (Advia 2120, Siemens Healthcare Nederland BV, Dan Haag, The Netherlands).

Haemolysis was determined as described previously by de Korte and colleagues.^19^ Briefly, cell‐free supernatants were obtained by centrifugation of the red cell concentrate at 12000 × *g* for 5 min followed by an additional centrifugation of the supernatant at 12000 × *g* for 5 min. Free Hb was determined by absorbance measurement of supernatant at 415 or 514 nm by a spectrophotometer (Eon plate reader, Bio Tek, Bad Friedrichshall, Germany), with correction for plasma absorption if necessary. Haemolysis was expressed as a percentage of total Hb present in the RBC after correction for haematocrit.

#### 2.3.3 Extracellular potassium

Whole, separated and washed blood samples were collected into a syringe and assayed for extracellular K+ using a blood gas analyser (RapidLab 1265, Siemens).

#### 2.3.4 RBC morphology

RBC morphology was determined after fixation of the cells with 1% glutaraldehyde. Using a light microscope (400× magnification) RBCs were visually analysed with a simplification of the scoring system described by Usry and colleagues^20^ as either discocytes (smooth biconcave discs, including echinodiscocytes without defined spicules) or echinocytes (crenated cells with defined spicules, including spherocytes). At least 200 cells were scored and the results are expressed as percentage echinocytes.

#### 2.3.5 Platelet morphology

Platelet morphology was assessed by Kunicki morphology scoring.^21^ The PC sample was fixed with 0.5% glutaraldehyde. Samples were analysed by phase contrast microscopy. The number of discs identified in a 100 cell count of fixed platelets under the microscope is multiplied by 4, spheres by 2, platelets with dendrites by 1, and balloons by 0, resulting in a maximal score of 400 for perfect discoid platelets.

#### 2.3.6 Platelet activation

WB was centrifuged (15 min, 210 × *g*) to produce platelet rich plasma. PLT activation was detected using a flow cytometer (LSRII‐HTS, BD Biosciences, Breda, the Netherlands)) after staining of PLTs with fluorescent CD62P‐FITC (P‐selectin, Beckman Coulter, Immunotech) as described before.^22^

#### 2.3.7 Total protein

Supernatant total protein was measured using the biuret method on Architect clinical chemistry analyser (Abbot, Abbot Park, IL, United States).

#### 2.3.8 Deformability

Referred to as the deformation index (DI), red blood cell deformability was defined as the ratio of the major axis length to the minor axis width. That is, the DI of a disc shaped red cell by definition equals 1. The DI was measured by means of an Automated Rheoscope and Cell Analyser (ARCA, Mechatronics Instruments, Hoorn, The Netherlands).^23^ This technology entails a thin layer of RBCs being sheared between two horizontal plates. One plate rotates with variable speed and distance from the other plate, resulting in variable, deforming force exerted on the red cells. Increasing force leads to elongation (deformation) of the red blood cells. This process is captured by a laser beam diffraction pattern, caught on camera and analysed computationally per individual cell. Findings are expressed as percentage of cells with DI <2.0 (non‐deformable cells) and DI with highest frequency.

#### 2.3.9 Thromboelastography properties

Thromboelastography (TEG) assays were performed using a TEG 5000 haemostasis system and plain cups and pins (Haemoscope Corp., Niles, IL, United States). WB samples were recalcified and the intrinsic coagulation pathway was stimulated with kaolin.^24^ Four values that represent clot formation were determined by this test: the reaction time (*R* value), the *K* value, the angle and the maximum amplitude (MA). The *R* value represents the time until the first evidence of a clot is detected. The *K* value is the time from the end of *R* until the clot reaches 20 mm and this represents the speed of clot formation. The angle is the tangent of the curve made as the *K* is reached and offers similar information to *K*. The MA is a reflection of clot strength.

### 2.4 Statistics

Results are expressed as mean values ± SD. Paired two‐sided *t*‐tests were performed to compare the WB measurements to the data acquired on the cellular and liquid components. Significance was defined as *p* < 0.05.

## 3 RESULTS

### 3.1 WB separation

**FIGURE 2:**
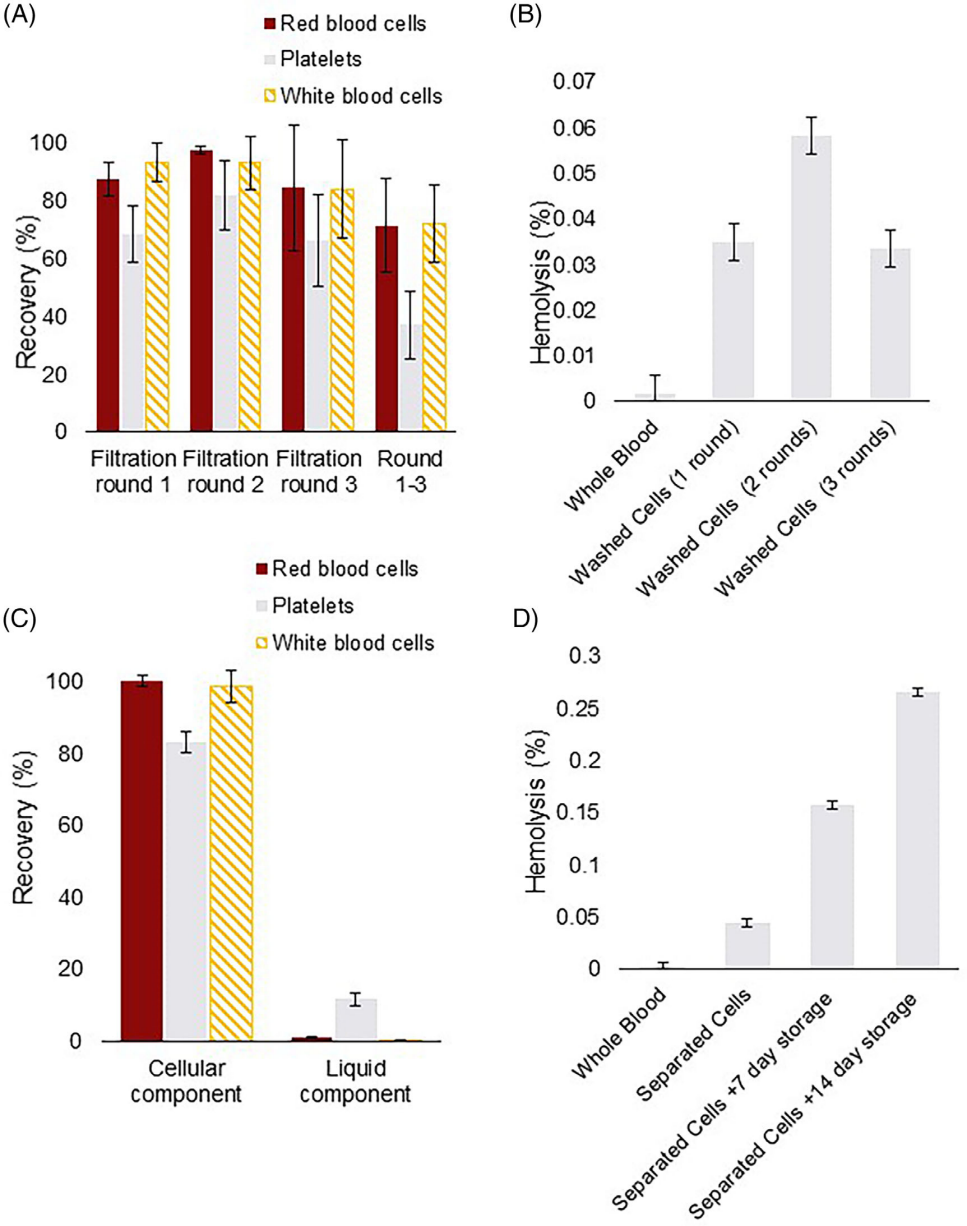
Cellular recovery. Error bars indicate SDs. (A) Cell salvage simulation mean percentual recoveries of red blood cells (RBCs), white blood cells (WBCs) and platelets (PLTs) in the cellular component per filtration round and over all three filtration rounds. (B) Mean percentage of haemolysis in the cellular components produced in the cell salvage simulation. (C) Mean percentual recovery of RBCs, WBCs and PLTs after undiluted separation into the liquid and cellular components. (D) Mean percentage of haemolysis in the cellular component produced in by undiluted separation

Three WB units (533 ± 5.9 ml) were separated using the HemoClear filter into a cellular component (392 ± 4.9 ml) and liquid component (219 ± 19 ml). Due to the priming of the system with saline, the combined volumes of the products obtained was higher than the volume of the WB. The recovery in the cellular component (including 110 ml of SAGM) of both WBCs (2.91 ± 0.49 × 10^9^ 99 ± 4.5%), RBCs (2.45 ± 0.25 × 10^12^ 100 ± 1.63%) and Hb (101 ± 1.5%) was around 100% (Figure 2C, Table S1). Cellular components also contained the larger fraction (101 ± 21.3 × 10^9^, 83 ± 3.0%) of the platelets, while the remainder (12 ± 1.9%) of platelets was found in the liquid component.

In line with RBC recovery, no significant difference was found between the haemolysis prior to (0.00 ± 0.01%), and after separation (0.04 ± 0.02%, *p* = 0.057). After 7 days of storage in SAGM at 2–6°C, haemolysis in the filtered cellular components (0.16 ± 0.02%, *p* = 0.006) had risen significantly, but remained below the requirement of <0.8%.^25^

#### 3.1.1 RBC deformability and morphology, and free potassium

**FIGURE 3:**
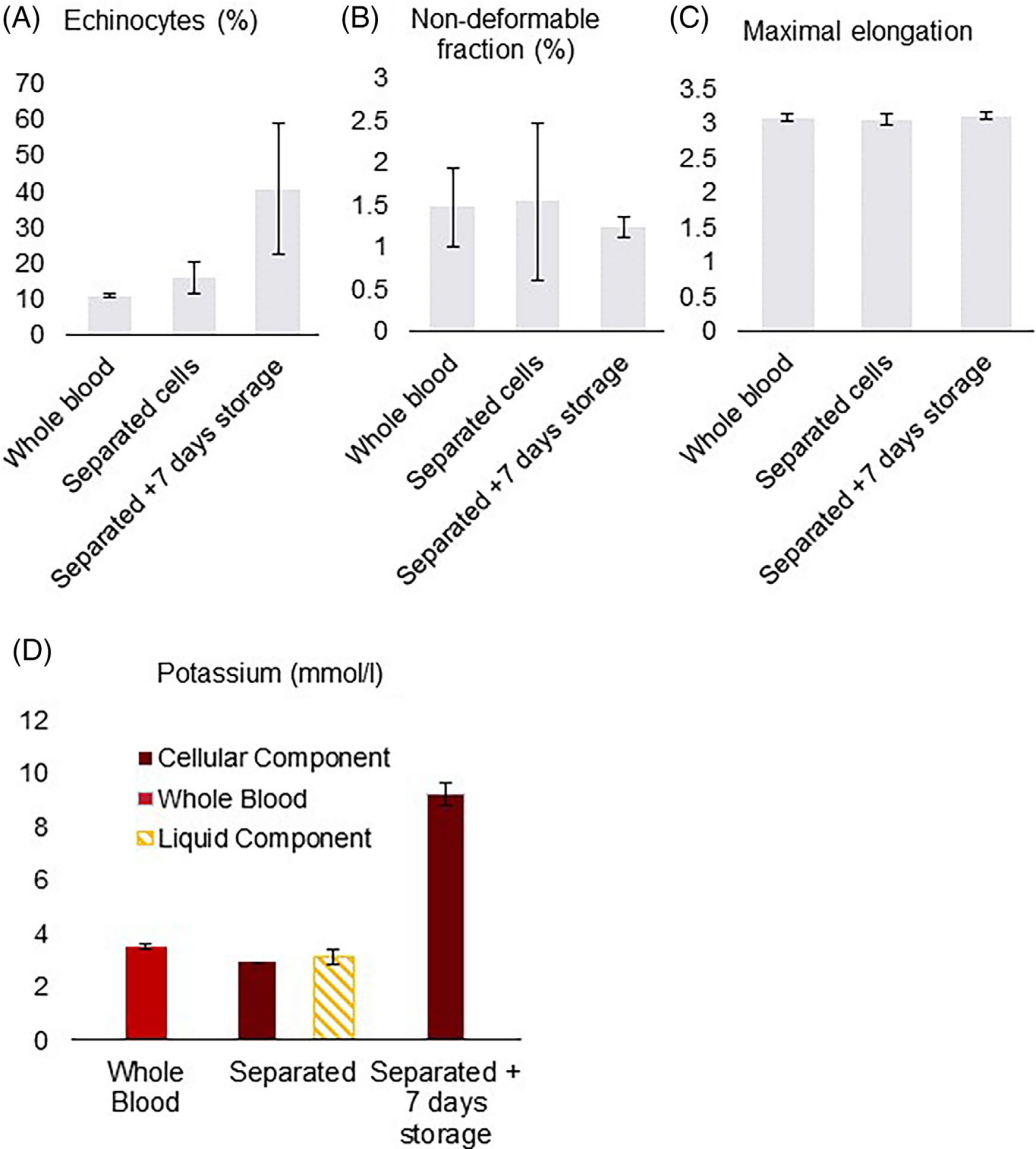
Red blood cell (RBC) deformability and morphology, and free potassium for the undiluted separation protocol. Mean values, error bars indicate SD. (A) Percentage of RBCs that are echinocytes in the undiluted separation protocol. (B) Percentage of non‐deformable RBCs in the undiluted separation protocol. (C) Maximal elongation in the undiluted separation protocol. (D) Concentration of free potassium in mmol/L

The non‐deformable RBC fraction as measured with ARCA, remained stable during separation (1.5 ± 0.46%, *p* = 0.881) and 7 days storage after the filtration (1.2 ± 0.12%, *p* = 0.434) (Figure 3B). Similarly, the RBC maximum elongation values were unaffected by the separation (3.1 ± 0.05, *p* = 0.664) and storage after processing (3.1 ± 0.05, *p* = 0.423) (Figure 3C).

Immediately after separation, 16 ± 4.4% of the RBCs were echinocytes, as compared to 11 ± 0.55% in the initial WB (*p* = 0.210) (Figure 3A). During storage of the filtered units, the number of echinocytes increased to 40.4 ± 18.3% (*p* = 0.109).

During the separation the total load of extracellular potassium remained stable. Storage of the filtered units resulted in a significant (*p* = 0.002) increase in extracellular potassium concentration from 2.9 mmol/L, immediately after filtration, to 9.2 ± 0.5 mmol/L after 7 days (Figure 3D).

#### 3.1.2 Platelet function, morphology and activation

**FIGURE 4:**
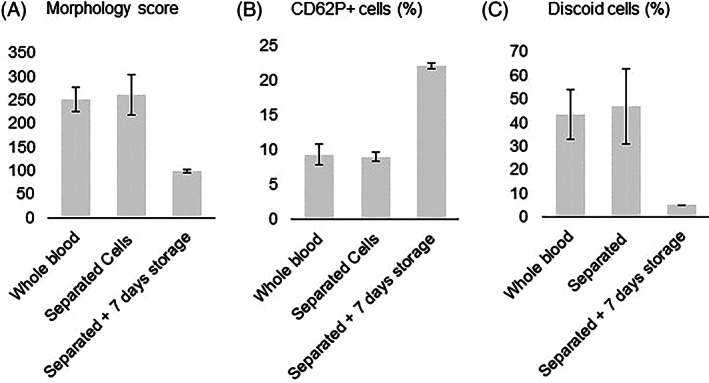
Platelet morphology and activation. Mean values, error bars indicate SDs. (A) Morphology score. (B) Percentage of platelets displaying CD62P, as an indication of platelet activation. (C) Percentage of discoid platelets

Immediately after separation of the WB, platelet morphology score (260 ± 44 vs. 250 ± 27 pre‐processed, *p* = 0.478), number of discoid cells (47 ± 16.% vs. 43 ± 10% pre‐processed, *p* = 0.529), and activation as measured by percentage CD62P positive PLT (9.0 ± 0.7% vs. 9.3 ± 1.5% pre‐processed, *p* = 0.560), were comparable to pre‐processed values (Figure 4). During cold storage, the morphology score (98.3 ± 2.9, *p* = 0.012) and percentage of discoid cells (5.0 ± 0.0%, *p* = 0.024) significantly declined compared to the WB, while platelet activation (22.0 ± 0.4%, *p* = 0.006) increased.

Platelet function was assessed using TEG as shown in Table 1. The *R* value (6.4 ± 0.5), that is, time until the first evidence of a clot, was reduced after filtration as compared to pre‐processed blood (9.2 ± 0.5, *p* = 0.005), indicating some activation due to the filtration. Other parameters of TEG, including *K* (*p* = 0.130), angle (*p* = 0.668) and MA (*p* = 0.125), were comparable for the filtered cells and WB. Indicating that separation has minimal influence on platelet functionality.

**TABLE 1:** Thromboelastographic data, means ± SD

	Whole blood	Separated cells	*p* value^*^	Separated + 7 day storage	*p* value^*^
*R* (min)	9.2 ± 0.5	6.4 ± 0.45	**0.005**	6.37 ± 0.40	**0.001**
*K* (min)	2.2 ± 0.3	2.4 ± 0.26	0.130	2.40 ± 0.26	**0.038**
Angle (°)	58.0 ± 3.2	57.7 ± 3.85	0.668	58.40 ± 2.02	0.695
MA (mm)	62.1 ± 5.6	56.3 ± 2.05	0.125	56.53 ± 3.61	0.334

	Whole blood	Washed cells	*p* value^*^	Healthy volunteers^26^	
*R* (min)	6.9 ± 0.63	6.1 ± 0.50	0.077	*R* (min)	3.8–9.8
*K* (min)	1.8 ± 0.37	2.9 ± 0.40	**0.002**	*K* (min)	0.7–3.4
Angle (°)	64.9 ± 4.3	53.9 ± 3.3	**0.003**	Angle (°)	47.8–77.7
MA (mm)	63.0 ± 2.7	42.5 ± 4.9	**<0.001**	MA (mm)	49.7–72.7

^*^ Statistical significance is indicated by *p* values in **bold**.

### 3.2 Washing of diluted WB

#### 3.2.1 Characteristics of cellular component

The washing procedure yielded a RBC recovery of at least 87 ± 6% (Hb recovery of at least 88 ± 6%), WBC recovery of at least 93 ± 7% and platelet recovery of at least 68 ± 10%.

Washing by one filtration round, two consecutive rounds or the entire washing protocol did not significantly increase haemolysis (Figure 2B).

#### 3.2.2 Washing efficiency

**FIGURE 5:**
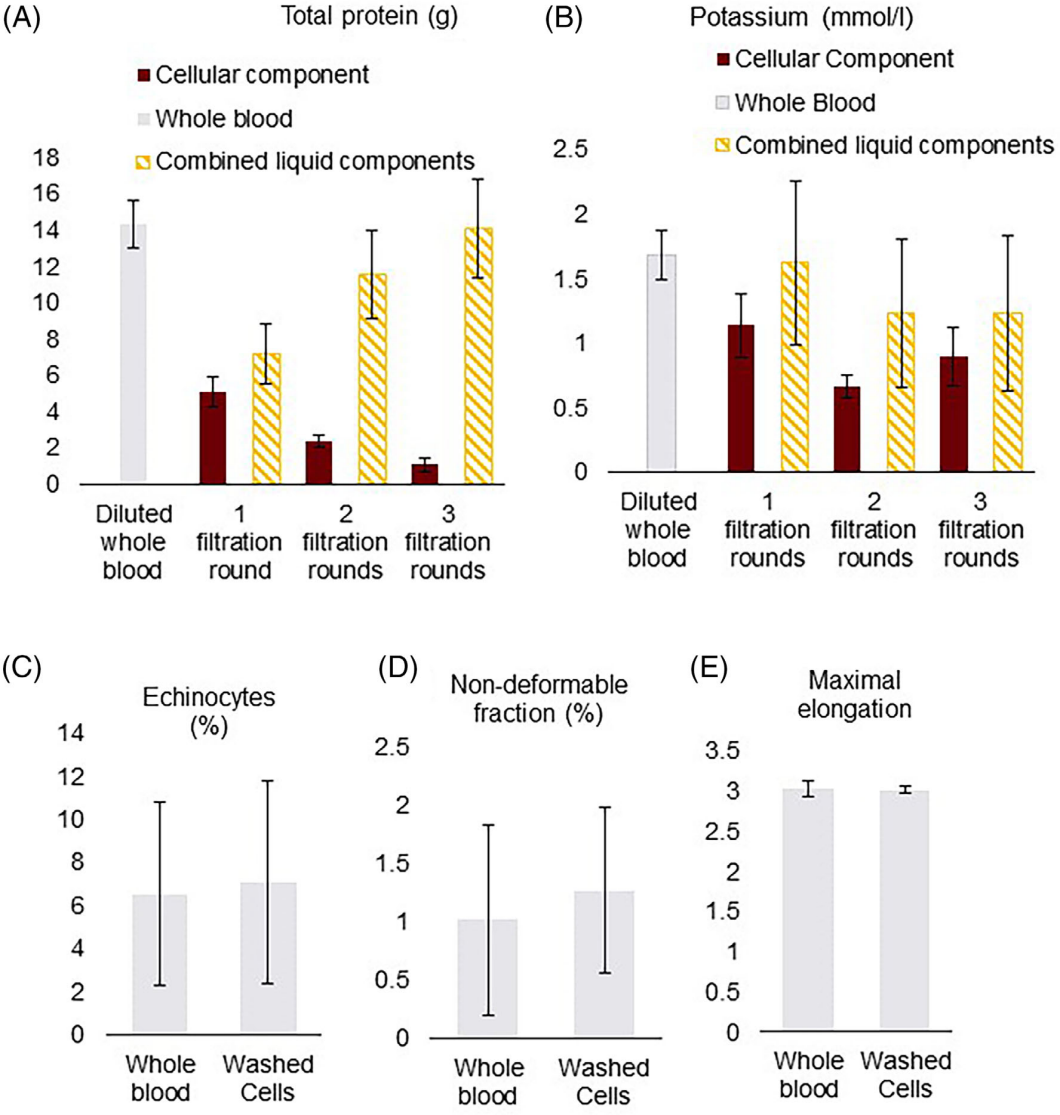
Cell salvage simulation washing effectiveness and red blood cell (RBC) morphology and deformability. Mean total protein in grams, error bars indicate SD. (A) Total protein load in grams. (B) Concentration of free potassium in mmol/L. (C) Percentage of RBCs that are echinocytes in the cell salvage simulation. (D) Percentage of non‐deformable RBCs in the cell salvage simulation. (E) Maximal elongation in the cell salvage simulation

Free total protein load was studied as an indication for the removal of extracellular solutes from the cellular component and washing effectivity (Figure 5A). Pre‐processed diluted WB units contained a mean free total protein load of 14 ± 1.3 grams. In an initial washing round about 35 ± 4.1% of the free total protein load was recovered in the cellular component (5.1 ± 0.84 g, *p* < 0.001), while 7.1 ± 1.6 grams ended up, washed out, in the liquid component. A subsequent second washing round of the cellular component further increased the free protein reduction to 84 ± 1.9%. After a third, concentration, filtration round, the free protein load in the cellular component was reduced by 93 ± 2.8%.

#### 3.2.3 RBC deformability and morphology

The non‐deformable RBC fraction as measured with ARCA, remained stable during the washing procedure (Figure 5D). Similarly, the RBC maximum elongation was unaffected by washing (Figure 5E). Washing also did not have a significant effect on the percentage of echinocytes. After two consecutive washing rounds 7.1 ± 4.7% of the RBCs were echinocytes (*p* = 0.808) (Figure 5C).

The total load of extracellular potassium remained stable throughout the washing protocol indicating no leakage of potassium from the red cells (data not shown). The potassium concentration in the cellular component was significantly reduced after the initial washing round (from 2.9 ± 0.35 to 1.1 ± 0.25 mmol/L, *p* = 0.012) (Figure 5B). In two consecutive washing rounds the potassium concentration in the cellular component was further reduced to 0.66 ± 0.09 mmol/L.

#### 3.2.4 Platelet function

Platelet function in the cellular component was assessed using TEG and are shown in Table 1. The *R* value, time until the first evidence of a clot, was similar in the washed RBCs (6.1 ± 0.5 min, *p* = 0.077) as compared to diluted WB (6.9 ± 0.6 min). The other parameters; *K*, angle and MA, were significantly altered in the washed RBCs but still within clinical functional values (Angle [°] 53.9 ± 3.3; MA 42.5 ± 4.9).^26^

## 4 DISCUSSION

The processing of WB units showed that the HemoClear device recovers high percentages of red blood cells, white blood cells and platelets. Cellular recoveries were higher in the separation protocol compared to the washing protocol. In the washing protocol, cellular recoveries in between washing rounds were measured while the filter and filtration lines still contained a fraction of the blood volume. This fraction of the blood cells was not included in the cellular count as determined in the blood bag. One way to correct for this seemingly lost volume is to measure the recoveries from the first washing round to the second round. This corrected calculation yielded RBC, WBC and platelet recoveries of 97.6 ± 9.6%, 93.0 ± 3.3% and 81.8 ± 12.9% respectively. (Figure 2A; Table S1). Red blood cell deformability and morphology, and platelet morphology and activation were not negatively affected in the neither separation nor washing protocol. Also the levels of free haemoglobin and potassium suggested minimal sublethal injury and haemolysis.

The unique cross‐flow microfiltration technology on which the studied device's mechanism is based, allows for both highly specific separation, washing and concentration of blood cells. The HemoClear could support centrifugal devices in separation of WB, and cell salvage. Moreover this device could be used to produce platelet‐rich RBCs.

### 4.1 Production of washed platelet‐rich red blood cells

Intraoperative cell salvage has been shown to increase platelet transfusion requirements.^27^, ^28^ Cell salvage is performed to recuperate red blood cells from shed blood. The autotransfusion device RBC washing procedures not only remove unwanted components, such as proinflammatory substances, but also eliminate platelets, coagulation factors and plasma proteins.

Second generation cell salvage devices are enhanced with platelet sequestration features that enable WB fractionation into RBCs and platelet‐rich plasma. However, evaluation of three autotransfusion devices showed that merely 50%–60% of platelets is recovered in the PRP with this enhanced function.^29^ With the HemoClear device there now is the possibility to salvage both washed RBCs and platelets. The produced platelet‐rich RBC product could be of high clinical value in the prevention of autotransfusion‐induced coagulopathies due to platelet loss. Especially in settings where centrifugal salvage devices are not affordable, not practical or not available this device should have added clinical value. Also in case of limited supply or availability of allogeneic platelets, the HemoClear device could prevent the need for it. Applicability of the HemoClear device in post‐operative cell salvage has already been reported in the field of cardiac surgery.^30^ It should be noted that an important factor expected to affect the adoption of a device like HemoClear is the cost‐effectiveness. The findings on the cost‐effectiveness of cell salvage have remained divided, being dependent on the medical setting and resources included.^31^, ^32^ As a potential pitfall to be considered cost‐effectiveness of the cell salvage procedure using HemoClear should be explored in a real‐world clinical setting.

### 4.2 Harvest of (convalescent) plasma

The total protein, free haemoglobin and potassium loads indicated that per HemoClear washing round about 65% of noncellular components is driven to the liquid component. Two consecutive washing rounds yielding 80%–90% of noncellular substances in the liquid component. Based on this finding we hypothesised usability of this device in the harvest of convalescent plasma. While finalising this manuscript, use of the device for the acquisition of anti‐COVID‐19 convalescent plasma was already studied by a group in Suriname.^33^ Bihariesingh‐Sanchit and colleagues applied a two round washing protocol, very similar to the washing protocol studied here, to isolate diluted convalescent plasma from recovered COVID19 patients in hospital.

Centrifugal apheresis is the main technology utilised in the collection of anti‐COVID convalescent plasma.^34^ Nevertheless, use of this technology in the emerging world is hindered by several barriers including lack of funds, no availability of apheresis kits and absence of technical expertise.^35^ Possibilities to use the device in production of convalescent plasma remain under study.

## CONFLICT OF INTEREST

Arno Nierich is the inventor of the HemoClear device, holds patent right to the device's technology and owns shares in HemoClear BV. Dion Osemwengie is employed by HemoClear BV.

## AUTHOR CONTRIBUTIONS

Dion Osemwengie designed the research study and wrote the manuscript. Richard Vlaar, Mya Go and Erik Gouwerok performed the research. Johan W. Lagerberg analysed the data. Johan W. Lagerberg, Arno P. Nierich and Dirk de Korte contributed to the research design and manuscript.

## Supporting information


**Table S1** Mean cell counts ± standard deviation.
