# Supplementary material for: Recovery of platelet‐rich red blood cells and acquisition of convalescent plasma with a novel gravity‐driven blood separation device
Source: Transfus Med. 2021 Nov 10;32(1):53–63. doi: 10.1111/tme.12830 (PMC9298860; doi:10.1111/tme.12830)
Supplement: Supplementary file 1 — Table S1 Mean cell counts ± standard deviation. [file TME-32-53-s001.docx]

# Supplemental Materials

Table S1. Mean cell counts ± standard deviation

| **Undiluted separation** |  |  |  |
| --- | --- | --- | --- |
|  | Whole Blood | Cellular component | Liquid component |
| RBC (10^12) | 2.44 ± 0.29 | 2.45± 0.25 | 0.02 ± 0.01 |
| PLT (10^9) | 122± 26.1 | 101 ± 21.3 | 14 ± 1.8 |
| WBC (10^9) | 1.25 ± 0.15 | 1.19 ± 0.14 | <0.01 |
| **Cell Salvage simulation** |  |  |  |
| *Filtration round 1* |  |  |  |
|  | Diluted WB | Cellular component | Liquid component |
| RBC (10^12) | 1.33 ± 0.10 | 1.16 ± 0.08 | 0.02± 0.03 |
| PLT (10^9) | 77 ± 21 | 53.89 ± 20.86 | 8.37± 8.13 |
| WBC (10^9) | 1.72 ± 0.45 | 1.63± 0.52 | <0.01 |
| *Filtration round 2* |  |  |  |
| RBC (10^12) |  | 1.13± 0.09 | 0.01± 0.01 |
| PLT (10^9) |  | 45.49± 22.55 | 6.36± 4.25 |
| WBC (10^9) |  | 1.52± 0.49 | <0.01 |
| *Filtration round 3* |  |  |  |
| RBC (10^12) |  | 0.96± 0.26 | 0.01± 0.01 |
| PLT (10^9) |  | 21.13± 7.00 | 4.68± 2.62 |
| WBC (10^9) |  | 1.24± 0.35 | <0.01 |
